# Deficits in Burrowing Behaviors Are Associated With Mouse Models of Neuropathic but Not Inflammatory Pain or Migraine

**DOI:** 10.3389/fnbeh.2018.00124

**Published:** 2018-06-28

**Authors:** Andrew J. Shepherd, Megan E. Cloud, Yu-Qing Cao, Durga P. Mohapatra

**Affiliations:** ^1^Washington University Pain Center, Washington University School of Medicine in St. Louis, St. Louis, MO, United States; ^2^Department of Anesthesiology, Washington University School of Medicine in St. Louis, St. Louis, MO, United States

**Keywords:** burrowing, pain, neuropathic pain, inflammatory pain, migraine

## Abstract

Burrowing, or the removal of material from an enclosed tube, is emerging as a prominent means of testing changes in a voluntary behavior in rodent models of various pain states. Here, we report no significant differences between male and female mice in terms of burrowing performance, in a substantially shorter time frame than previous reports. We found that the color of the burrow tube affects the variability of burrowing performance when tested in a lit room, suggesting that light aversion is at least a partial driver of this behavior. Spared nerve injury (SNI; as a model of neuropathy) impairs burrowing performance and correlates with enhanced mechanical sensitivity as assessed by von Frey filaments, as well as being pharmacologically reversed by an analgesic, gabapentin. Loss of the SNI-induced burrowing deficit was observed with daily testing post-surgery, but not when the testing interval was increased to 5 days, suggesting a confounding effect of daily repeat testing in this paradigm. Intraplantar complete Freund’s adjuvant (as a model of inflammatory pain) and systemic nitroglycerin (as a model of migraine-like symptoms) administration did not induce any burrowing deficit, indicating that assessment of burrowing behavior may not be universally suitable for the detection of behavioral changes across all rodent pain models.

## Introduction

Pre-clinical animal models of painful pathologies have historically relied upon stimulus-evoked assessment of pain sensitivity, such as the Hargreaves test for thermal sensitivity and von Frey filament-based assessment of mechanical sensitivity. However, the insufficient translation of pre-clinical findings into clinical practice has cast doubt on the validity of such tests (Barrot, 2012). With the goal of enhancing the translational potential of preclinical findings in pain research, newer assays have been developed that assess changes in voluntary or operant behaviors as surrogate pain measures, particularly those behaviors that are depressed in animals experiencing pain (Langford et al., 2010; Negus et al., 2010; Mogil, 2015; Harte et al., 2016; Sheahan et al., 2017; Shepherd and Mohapatra, 2018).

One such example is burrowing, an ancient, adaptive behavior conserved across many rodent species, and one in which various laboratory strains of mice and rats spontaneously engage. As such, it is considered to represent a correlate of so-called “activities of daily living” in humans—tasks that are essential to satisfactory quality of life and are often impeded by pain (Deacon et al., 2001; Deacon, 2006; Jirkof, 2014). Various groups have showed that burrowing is depressed in neuropathic and inflammatory pain states, in both mice and rats (Jirkof et al., 2010; Andrews et al., 2012; Huang et al., 2013; Lau et al., 2013; Rutten et al., 2014; Bryden et al., 2015; Gould et al., 2016; Muralidharan et al., 2016; Wodarski et al., 2016; Das et al., 2017). However, there are several key variables that remain to be characterized, namely sex, tube color/lighting, uniform burrowing materials, and the relative sensitivity of burrowing assays across different pain models.

We developed a standardized version of the burrowing assay using a substrate identical to that used in the home cage environment, coupled with a relatively brief assay duration. Our study shows that male and female mice of multiple backgrounds displace similar amounts of bedding after 15 min of access to a bedding-filled tube, with relatively little inter-individual variability. The spared nerve injury (SNI) model of traumatic neuropathy produces a deficit in this burrowing behavior that is correlated with evoked mechanical sensitivity (as assessed by von Frey filament testing). Gabapentin, a known analgesic for neuropathic pain, reverses this SNI-induced suppression of burrowing behavior, suggesting it to be pain-related. Models of inflammatory pain and migraine-like condition do not show burrowing deficits in our hands, suggesting that although the assay is rapid, inexpensive and reproducible, it may not have the same universality as a surrogate measure of pain when compared to more traditional, evoked measures.

## Materials and Methods

### Animals

This study was carried out in accordance with the recommendations of the “NIH Guidelines for the Care and Use of Laboratory Animals”. The protocol was approved by the “Institutional Animal Studies Committee of Washington University”. Every effort was made to minimize the number of mice used and their suffering. Mice were maintained on a 12:12 light: dark cycle (light from 06:00 h to 18:00 h) with access to food and water *ad libitum*. Male and female C57BL/6J (Stock No: 000664) and FVB/NJ mice (Stock No.: 001800, both Jackson Laboratories) were used between 8 weeks and 14 weeks of age for all experiments. Mice were socially housed segregated by sex, five to a cage, containing $\frac{1}{8}$ inch corncob bedding (Teklad-Envigo, Cat. No. 7092) and cotton fiber-based nesting material. Each cage of mice contained both groups of animals, i.e., those that were assigned to the control or experimental (nerve injury, Complete Freund’s Adjuvant (CFA), nitroglycerin (NTG)) interventions. The numbers of animals in individual experimental (and respective control) groups are specified in the figures and figure legends. Experimenters were blinded to mouse sex, details of injected agents, and injection laterality during experimental data collection/recording and subsequent analysis.

### Burrowing Tubes

**Figure 1:**
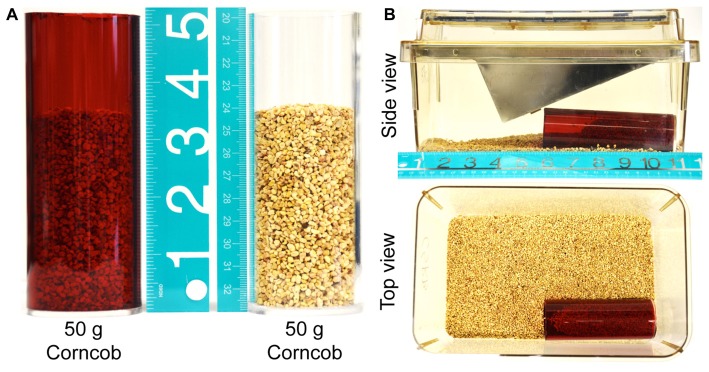
Experimental setup. **(A)** Red and transparent acrylic tubes filled with 50 g of corncob bedding, illustrating the proportion of the tube that is occupied by bedding material at the start of the experiment. **(B)** The bedding-filled tube is placed in the cage as depicted, with the wire lid and plastic cage top in place. The food hopper is filled with standard mouse chow to replicate the home cage environment more closely.

Acrylic tubing (red; SKU: 23396, transparent; SKU: 23388, outer diameter 2 inch or 51 mm; inner diameter 1.75 inch or 44 mm) was purchased from TAP Plastics Inc., (Stockton, CA, USA). 5 inch (125 mm) lengths of tubing were cut and one end was sealed with a transparent acrylic circle-lid (2 inch or 51 mm diameter × 0.118 inch/3 mm thick; SKU: 01902) and acrylic adhesive (Figure 1A). This specific length of burrowing tubes were based on half of the mouse cage length, in order to provide sufficient distance from the opening of the tube for effective displacement of burrowing/bedding material (Figure 1B). Tubes were washed with warm water and detergent and thoroughly rinsed prior to first use, and rinsed with distilled water and air-dried in between experimental time points. The tubes were numbered and the same tube was used for a particular mouse over the duration of an experiment.

### Burrowing Assay

Mice were acclimatized to the specific burrowing tube (red or transparent) once on the day before the burrowing assay was to be performed (for 1 h), and again on the day of testing (for 30 min). For acclimatization an empty tube was placed into the home cage, such that all five mice in the cage were exposed to the tube. All mice were observed to voluntarily enter the tube within 10–15 min (data not shown). Although we did not test this specific issue, our initial exposure of mice to a burrowing tube in a socially-housed home cage may be an important means of effectively habituating these mice, as others have theorized that such a setup allows social facilitation of investigation/entry into the tube (Deacon, 2006).

On the day of testing, a separate cage was prepared for each mouse to be tested individually (Figure 1). An acrylic tube was filled with 50 g of the same corncob bedding used in the home cage and placed in one corner, parallel to the long walls of the cage. Unless otherwise stated, mice were then transferred to these cages for 15 min, at which point the mouse was returned to the home cage and the bedding remaining in the tube was weighed. The burrowing activity was calculated by subtracting the weight of bedding present at the end of the experiment from the starting weight (50 g), and expressing the proportion of bedding that had been displaced as a percentage. In order to minimize any potentially confounding effects of differing olfactory cues, approximately 5 g of bedding from the home cage was transferred to the testing cage immediately prior to first testing. In addition, all bedding from a particular test cage was stored for re-use in a re-sealable plastic bag between testing.

### von Frey Filament-Based Assessment of Hindpaw Mechanical Sensitivity

Mechanical sensitivity was measured as described previously (Mickle et al., 2015; Shepherd and Mohapatra, 2018; Shepherd et al., 2018) using a range of eight von Frey monofilaments (0.04–2 g; Stoelting Co., Wood Dale, IL, USA) applied to the lateral aspect of the plantar surface of the hindpaw. Mice were habituated to a wire mesh platform covered by a Plexiglas box 24 h prior to testing and for 15 min immediately before testing. Tests were performed starting with five presentations of the lowest filament strength (0.04 g), followed by application of progressively greater filament strengths in the same manner until the filament with maximum strength (2 g) was reached. Each filament was applied to an individual mouse hind paw five times, and the number of paw withdrawal responses was recorded as a percent response. The interval between successive presentations of the same filament was ≥5 s or until behavior evoked by presentation of the filament had ceased. The number of withdrawal responses was recorded for each hindpaw across all eight filaments (with a ≥5-min interval between testing of any one mouse with a particular filament). To assess changes in paw withdrawal response to the whole range of filaments over a particular testing period, the area under the curve (AUC) value was calculated from the filament-response curve for each animal. Using these values, the average AUC for each treatment group was calculated, similar to the analysis method described earlier (Banik et al., 2006) with minor modifications, as detailed previously (Mickle et al., 2015; Shepherd and Mohapatra, 2018; Shepherd et al., 2018).

### Spared Nerve Injury of Experimental Neuropathy in Mice

SNI was performed as described previously (Decosterd and Woolf, 2000; Shepherd and Mohapatra, 2018). Briefly, mice were anesthetized with isoflurane, and an incision was made proximal to the knee, such that the *biceps femoris* muscle was exposed. The muscle layers were separated by blunt dissection to access the branches of the sciatic nerve. The common peroneal and tibial branches were ligated with 6–0 silk suture, and approximately 1 mm of nerve was removed distally. The sural branch remained untouched. Following closure of the incision and administration of post-operative analgesia, mice were allowed to recover in a clean cage warmed from below with a temperature-controlled heating pad. Sham operation controls underwent the same procedure, with the exception that the sciatic nerve wasn’t ligated or sectioned. Post-operative testing on these mice began no fewer than 5 days after surgery, in order to allow complete recovery from surgery. For experiments with gabapentin injection in mice with sham or SNI surgery, systemic injection of gabapentin (10 mg/kg in saline, i.p.; Sigma-Aldrich, St. Louis, MO, USA) or saline as a vehicle control was performed 1 h prior to behavioral testing.

### Complete Freund’s Adjuvant-Based Model of Inflammatory Pain

Mice were injected with CFA as described previously (Shepherd and Mohapatra, 2018). Briefly, mice were manually restrained with a small cloth such that one hindlimb could be exposed and stabilized, plantar surface facing up. Ten microliters of a 1 mg/ml suspension of CFA (Sigma-Aldrich, St. Louis, MO, USA), or saline were injected to mice into the plantar surface of one hindpaw using a 0.3 ml insulin syringe.

### Nitroglycerin-Based Model of Migraine

Mice received repetitive intraperitoneal injections of nitroglycerin (NTG; 10 mg/ml/kg in saline, freshly diluted from 10% stock, Copperhead Chemical) or vehicle (1% propylene glycol in saline, 10 ml/kg) every 2 days for five times as described previously (Pradhan et al., 2014). Hindpaw mechanical threshold was measured with von Frey Filaments 2 days before the first injection and 2 days after the last injection to verify the establishment of NTG-induced mechanical hypersensitivity (data not shown). In female mice, burrowing assays were performed 1 day before the first injection and 2 days after each injection. In male mice, burrowing assays were performed 1 day before the first injection and 2 days after the third and last injections. On each test day, mice were tested with the transparent and red tubes in the morning.

### Statistical Analysis

Data are presented as mean percent bedding displaced or mechanical sensitivity ± SEM. No animals were excluded from testing, based their baseline performance, and no data were normalized to baseline values. Two-way ANOVA with Tukey’s or Bonferroni’s *post hoc* test or three-way ANOVA with Tukey’s *post hoc* was performed where appropriate. Pearson’s correlation analysis was performed to calculate the degree of correlation between mechanical sensitivity and burrowing activity. *p* < 0.05 for each comparison group was considered statistically significant. All analysis was performed using GraphPad Prism 7.0 (GraphPad Software Inc., La Jolla, CA, USA).

## Results

### Bedding Displacement (Burrowing) Behavior in Mice Is Dependent Upon Test Duration, Tube Color and Circadian Conditions

**Figure 2:**
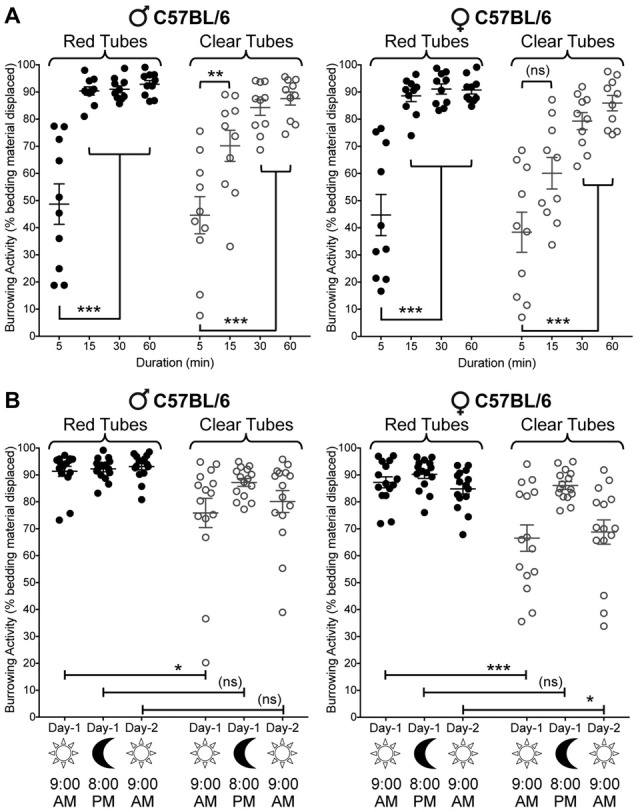
Effect of sex, tube color, assay duration and time of day/lighting on bedding displacement. **(A)** Male C57BL/6 mice (left) display a time-dependent increase in bedding material displacement, with saturating displacement occurring after 15 min. The variability of bedding displacement is increased in experiments using transparent tubes (empty circles) vs. red tubes (filled circles). Female C57BL/6 mice (right) show similar displacement kinetics to those seen in males. Mean ± SEM (*n* = 15/group). **(B)** Male C57BL/6 mice (left) exhibit similar bedding displacement from red tubes when tested during the light cycle (9:00 AM—lights ON; 15 min) and dark cycle (8:00 PM—lights OFF; 15 min). Clear tubes are associated with greater variability in bedding displacement, but only during the light cycle. Female C57BL/6 mice (right) also exhibit this light/time-dependent difference in displacement variability with transparent tubes. Data are presented as individual animal data points, with mean ± SEM marked (*n* = 15/group). *p < 0.05, **p < 0.01, ***p < 0.001 and “ns”-not significant for indicated group comparisons, three-way ANOVA with Tukey’s *post hoc* correction.

Before we began investigating the effects of various pain-related models on burrowing behavior, we first characterized the impact of several key variables on burrowing performance. Mice (C57BL/6J) were placed into cages containing a translucent red tube filled with 50 g of bedding, and removed after 5, 15, 30 or 60 min. These experiments were performed between 08:00 AM and 11:00 AM (2–5 h of the light cycle). At 5 min, bedding displacement reached an average of 48.7 ± 7.5%, with a large range of individual values (range: 19%–77%, *n* = 10; Figure 2A, left panel). At 15 min, the average displacement increased to 90.4 ± 1.5%, with much lower variability (range: 81%–98%, *n* = 10). Allowing access to the tube for a total of 30 min or 60 min had little additional impact on the mean amount of bedding removed, or the inter-individual variability (91.0 ± 1.3% at 30 min, 92.9 ± 1.4% at 60 min; Figure 2A, left panel). Interestingly, when the tubes used were transparent, the amount of bedding displaced at 15 min was lower (44.6 ± 6.7%), and the variability was higher (range: 8%–76%, *n* = 10), suggesting that the perceived light intensity within the tube could have an impact on burrowing speed and variability. Similar results were observed in both male and female C57BL/6J mice (Figure 2A). Significance was observed in three-way ANOVA for the following factors: Duration of test: (*F*_(3,144)_ = 83.79, *p* < 0.0001), tube color (*F*_(1,144)_ = 23.45, *p* < 0.0001), time × tube color (*F*_(3,144)_ = 311, *p* = 0.0084), Thus, for logistical reasons, a testing period of 15 min was adopted in subsequent experiments.

We next wanted to verify whether the point in the sleep-wake cycle at which we tested mice, or the lighting in the testing room could influence burrowing behavior. When testing mice in red tubes (the interior of which is perceived as relative darkness by mice), bedding material displacement was not influenced by the time of day/lighting conditions (9:00 AM—lighting ON; 8:00 PM—lighting OFF; Figure 2B). As established in Figure 2A, transparent tubing is associated with highly variable burrowing behavior when the experiment is run in a normally lit room during daylight hours (9:00 AM—lighting ON). However, subsequent testing with transparent tubing on the same day during the evening (8:00 PM—lighting OFF) in a darkened testing room, markedly reduces this variability, increasing the average bedding displacement (mean: 75.9 ± 5.4% in the light, range: 20%–95%; mean: 87.2 ± 1.5% in darkness, range: 77%–95%; Figure 2B). Importantly, this darkness-associated increase in bedding displacement was reversed when the experiment was repeated the following morning (9:00 AM—lights ON; Figure 2B, left panel). Significance was observed in three-way ANOVA for the following factors: time of test: (*F*_(2,168)_ = 9.376, *p* = 0.0001), sex (*F*_(1,168)_ = 11.84, *p* = 0.0007), tube color (*F*_(1,168)_ = 50, *p* < 0.0001) and test time × tube color (*F*_(2,168)_ = 5.29, *p* = 0.0059). Again, similar observations were made in both male and female mice (Figure 2B). Given that the time of the day and/or room lighting influence burrowing activity when transparent tubes are used, but not red tubes, these data suggest that perceived light intensity within the tube presumably influences burrowing behavior in mice.

### Burrowing Behavior in Mice Following SNI Is Negatively Correlated With Cutaneous Mechanical Sensitivity

**Figure 3:**
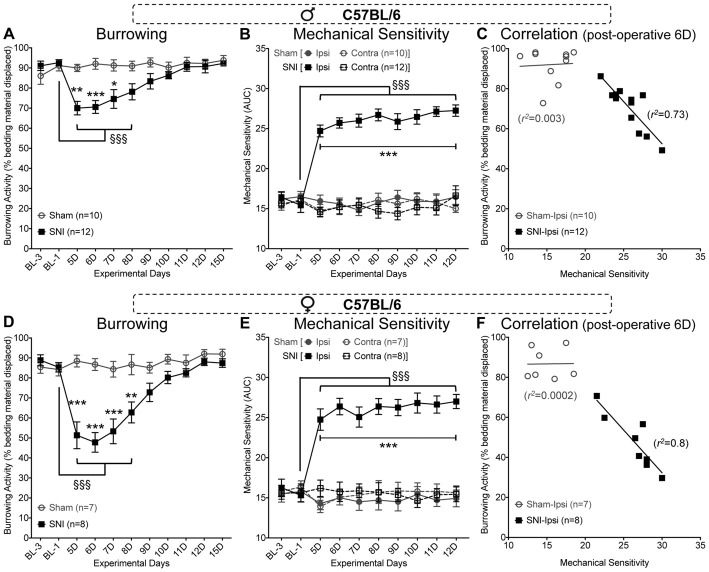
Spared nerve injury (SNI) suppresses bedding displacement, referred to as burrowing behavior in C57BL/6 mice. SNI suppresses burrowing behavior relative to sham-surgery controls on post-operative days 5–7 in male **(A)** and days 5–8 in female **(D)** mice. Hindpaw mechanical sensitivity as assessed by von Frey filament testing is significantly elevated after SNI surgery and remains elevated through post-operative day 12 in male **(B)** and female **(E)** mice. At post-operative day 6, the extent of ipsilateral hindpaw mechanical sensitivity is significantly negatively correlated with the extent of burrowing behavior in male **(C)** and female **(F)** mice with SNI, but not sham-surgery controls. Data in **(A,B,D,E)** are presented as mean ± SEM. For **(A,D)**, ^§§§^*p* < 0.001 for values in SNI group experimental day-5 (5D) onwards, compared to BL-1 control time point, and *p < 0.05, **p < 0.01, ***p < 0.001 for individual testing days in SNI vs. sham operation controls, two-way ANOVA with Tukey’s *post hoc* correction. For **(B,E)**, ^§§§^*p* < 0.001 for values in SNI-ipsi group experimental day-5 (5D) onwards, compared to BL-1 control time point, and ***p < 0.001 for individual testing days in SNI-ipsi vs. SNI-contra, sham-ipsi and sham-contra operation controls, three-way ANOVA with Tukey’s *post hoc* correction.

With the establishment of experimental conditions that resulted in displacement of 80%–90% of bedding from red tubes in 15 min testing period (Figure 2), we next tested whether such behavior could be altered under neuropathic conditions. In C57BL/6J male mice subjected to unilateral SNI, we observed a significant reduction in bedding displacement using red tubes tested during morning hours on post-operative days 5–7 (Figure 3A). This effect gradually diminished over the remainder of the testing period, such that SNI mice were indistinguishable from sham surgery controls on post-operative days 10–15. Data were analyzed using two-way ANOVA to compare the effect of SNI surgery (vs. sham surgery) and the time course (post-surgery time points vs. pre-surgery baseline measurements). SNI surgery yielded an “F” ratio of *F*_(1,20)_ = 6.926, *p* = 0.016. The time course comparison yielded an “F” ratio of *F*_(10,200)_ = 8.471, *p* < 0.0001. The interaction between factors was significant (*F*_(10,200)_ = 8.351, *p* < 0.0001).

Assessment of mechanical sensitivity using von Frey filament-based assay in the same cohort of mice displayed the typical, persistent mechanical hypersensitivity in the lateral aspect of the ipsilateral hindpaw (Figure 3B). Mice were tested in this assay, immediately following the completion of burrowing behavior assessment during the morning hours. Significance was observed in three-way ANOVA for the following factors: experimental days: (*F*_(9,360)_ = 4.805, *p* < 0.0001), surgery (*F*_(1,360)_ = 218, *p* < 0.0001), paw laterality (*F*_(1,360)_ = 311, *p* < 0.0001), experimental days × surgery (*F*_(9,360)_ = 6.608, *p* < 0.0001), experimental days × paw laterality (*F*_(9,360)_ = 7.579, *p* < 0.0001), surgery × paw laterality (*F*_(1,360)_ = 262.5, *p* < 0.0001) and experimental days × surgery × paw laterality (*F*_(9,360)_ = 8.344, *p* < 0.0001). This suggests that evoked mechanical hypersensitivity induced by SNI does persist, but this hypersensitivity is not necessarily paralleled in burrowing behavior after repeated daily testing. Interestingly, at post-SNI time points where burrowing behavior is still depressed, the degree of hindpaw (ipsilateral) mechanical hypersensitivity that SNI mice exhibit in von Frey testing is highly correlated with bedding displacement (Pearson *r* coefficient = −0.854 at post-operative day 6, *n* = 12, *p* = 0.0004; Figure 3C).

These observations were reproduced in female C57BL/6J mice (Figures 3D–F). In the burrowing assay, SNI vs. sham surgery yielded an “F” ratio of *F*_(1,13)_ = 23.14, *p* = 0.0003. The time course comparison yielded an “F” ratio of *F*_(10,130)_ = 13.45, *p* < 0.0001. The interaction between factors was significant (*F*_(10,130)_ = 10.86, *p* < 0.0001). SNI induced a marginally greater depression of burrowing behavior in female mice vs. male mice on post-operative days 5 (*p* < 0.01), 6, 7 (*p* < 0.001) and 8 (*p* < 0.05), *F*_(1,18)_ = 11.46, *p* = 0.0033 for SNI surgery in males vs. females. The time course comparison yielded an “F” ratio of *F*_(10,180)_ = 37.69, *p* < 0.0001. The interaction between factors was significant (*F*_(10,180)_ = 3.371, *p* < 0.0005).

As with male C57BL/6J mice, von Frey testing showed a sustained elevation of ipsilateral hindpaw mechanical sensitivity in female SNI mice throughout the testing period. Significance was observed for the following factors: experimental days: (*F*_(9,240)_ = 4.217, *p* < 0.0001), surgery (*F*_(1,240)_ = 195.4, *p* < 0.0001), paw laterality (*F*_(1,240)_ = 136.4, *p* < 0.0001), experimental days × surgery (*F*_(9,240)_ = 6.964, *p* < 0.0001), experimental days × paw laterality (*F*_(9,240)_ = 3.988, *p* < 0.0001), surgery × paw laterality (*F*_(1,240)_ = 182.6, *p* < 0.0001) and experimental days × surgery × paw laterality (*F*_(9,240)_ = 4.615, *p* < 0.0001). As with male mice, the degree of mechanical sensitivity was also negatively correlated with the amount of bedding displaced (Pearson *r* coefficient = −0.895 at post-operative day 6, *n* = 8, *p* = 0.003; Figure 3F). These data indicate that there is a close association between the suppression of burrowing behavior and the extent of evoked mechanical hypersensitivity.

**Figure 4:**
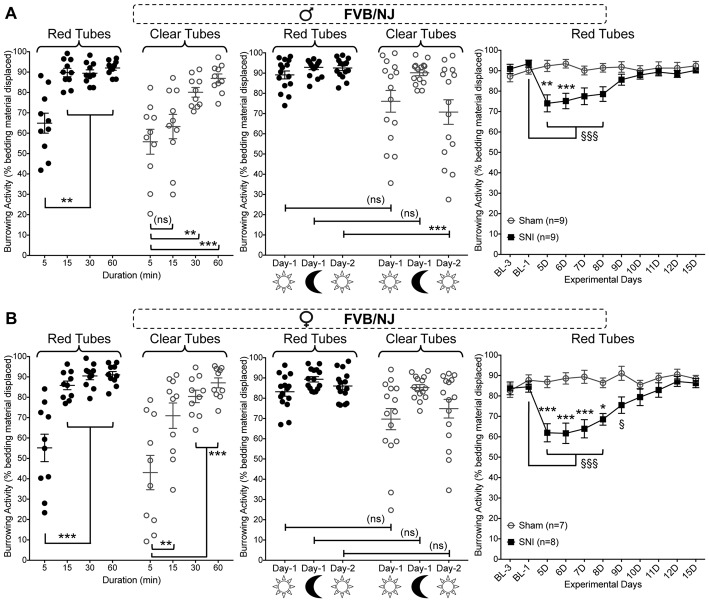
Effect of sex, tube color, assay duration and time of day/lighting on bedding displacement, as well as SNI-induced deficits in burrowing behavior in FVB/NJ mice. Both male **(A)** and female **(B)** mice display a time-dependent increase in bedding material displacement (left panel), with saturating displacement occurring after 15 min. The variability of bedding displacement is increased in experiments using transparent tubes (empty circles) vs. red tubes (filled circles). A similar bedding displacement from red tubes is observed in mice when tested during the light cycle (9:00 AM—lights ON; 15 min) and dark cycle (8:00 PM—lights OFF; 15 min). Clear tubes are associated with greater variability in bedding displacement, but only during the light cycle (middle panels). SNI significantly suppresses burrowing behavior relative to sham-surgery controls on post-operative days 5–6 in male **(A)** and days 5–8 in female **(B)** mice (right panels). Data in the left and middle graphs in both panels are presented as individual animal data points, with mean ± SEM marked, and as mean ± SEM for right graphs in both panels. For left and middle graphs in both panels **p < 0.01, ***p < 0.001 and “ns”-not significant for indicated group comparisons, three-way ANOVA with Tukey’s *post hoc* correction. For right graphs in both panels ^§^*p* < 0.05, ^§§§^*p* < 0.001 for values in SNI group experimental day-5 (5D) onwards, compared to BL-1 control time point, and *p < 0.05, **p < 0.01, ***p < 0.001 for individual testing days in SNI vs. sham operation controls, two-way ANOVA with Tukey’s *post hoc* correction.

We next tested if such neuropathy-induced deficits in burrowing behavior could be observed in a mouse strain other than C57BL/6J. We used the FVB/NJ mouse strain, which is commonly utilized for generating genetically-modified mice. This mouse strain exhibited a similar burrowing activity time course, both in red and clear tubes, as compared to C57BL/6J mice (Figure 4A, left panel). Also, there were no sex differences in the burrowing time course for FVB/NJ mice (Figures 4A,B, left panels). Furthermore, FVB/NJ mice exhibit similar circadian- and light/dark-dependance of burrowing activity and/or variability in both males and females (Figures 4A,B, middle panels). Induction of SNI in FVB/NJ mice led to a significant deficit in burrowing activity on 5 and 6 days post-surgery (in males), and 5 through 8 days (females), which subsequently returned to the levels exhibited by sham-operated mice (Figures 4A,B, right panels). Data were statistically analyzed using two-way ANOVA to compare the effect of SNI surgery (vs. sham surgery) and the time course (post-surgery time points vs. pre-surgery baseline measurements). SNI surgery yielded an “F” ratio of *F*_(1,16)_ = 7.275, *p* = 0.016. The time course comparison yielded an “F” ratio of *F*_(10,160)_ = 4.379, *p* < 0.0001. The interaction effect was significant (*F*_(10,160)_ = 6.839, *p* < 0.0001). These observations indicate that at least two distinct strains of mice exhibit similar burrowing behaviors at baseline and post-injury.

### SNI-Induced Suppression of Burrowing Activity Persists With a Greater Testing Interval, and Can Be Reversed by Gabapentin

**Figure 5:**
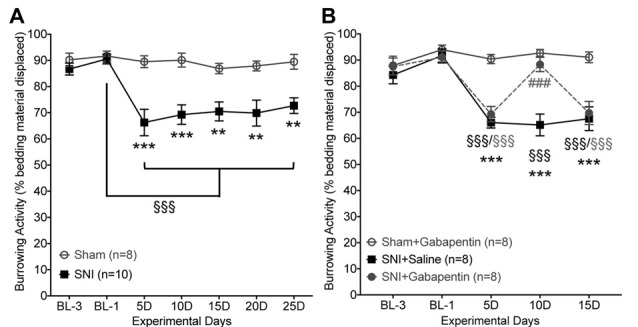
SNI-induced suppression of burrowing behavior is reversed by gabapentin. **(A)** With an inter-test interval of 5 days, burrowing behavior is suppressed in SNI vs. sham-surgery control mice from post-operative days 5–25. **(B)** SNI-induced suppression of burrowing behavior is acutely reversed by gabapentin (10 mg/kg, i.p.). Data are presented as mean ± SEM. ^§§§^*p* < 0.001 for values in SNI groups experimental day-5 (5D) onwards, compared to their BL-1 control time point, **p < 0.01, ***p < 0.001 for SNI vs. respective sham operation controls, and ^###^*p* < 0.001 for SNI+gabapentin vs. SNI+saline, two-way ANOVA with Bonferroni’s *post hoc* correction.

We reasoned that the gradual reversal of the SNI-induced deficit in burrowing behavior might be due to the use of a daily testing paradigm. Accordingly, we repeated the burrowing behavior experiments in a separate cohort of sham/SNI mice with an inter-test interval of 5 days (Figure 5A). In contrast to the gradual return to normal burrowing behavior seen with daily testing of SNI mice (Figures 3A,D), maintaining a 5-day interval between testing resulted in a persistent decrease in bedding displacement in SNI mice vs. sham surgery controls (Figure 5A). Factor “surgery”: (*F*_(1,16)_ = 22.98, *p* < 0.0001), “time” (*F*_(6,96)_ = 7.701, *p* < 0.0001), interaction: (*F*_(6,96)_ = 5.003, *p* = 0.0002).

In order to validate a change in a voluntary behavioral assessment as pain-related, it is necessary to establish whether the change in behavior can be reversed by an established analgesic. To that end, we show that SNI-induced suppression of burrowing behavior could be acutely reversed by systemic administration of gabapentin (10 mg/kg, i.p.) 1 h prior to testing (Figure 5B). Injection of saline in SNI mice had no effect on burrowing behavior, and gabapentin did not influence burrowing behavior of sham-surgery controls (Figure 5B). Factor “surgery/drug”: (*F*_(2,21)_ = 16.42, *p* < 0.0001), “time” (*F*_(4,84)_ = 24.58, *p* < 0.0001), interaction: (*F*_(8,84)_ = 9.244, *p* < 0.0001). As expected, this dose of gabapentin also led to complete attenuation of hindpaw mechanical hypersensitivity (not shown). This suggests that a greater interval between testing of burrowing behavior enables observation of ongoing behavioral suppression, and that an analgesic can acutely reverse this suppressed burrowing behavior back to control levels.

### Models of Inflammatory Pain and Migraine Are Not Associated With Suppression of Burrowing Behavior

**Figure 6:**
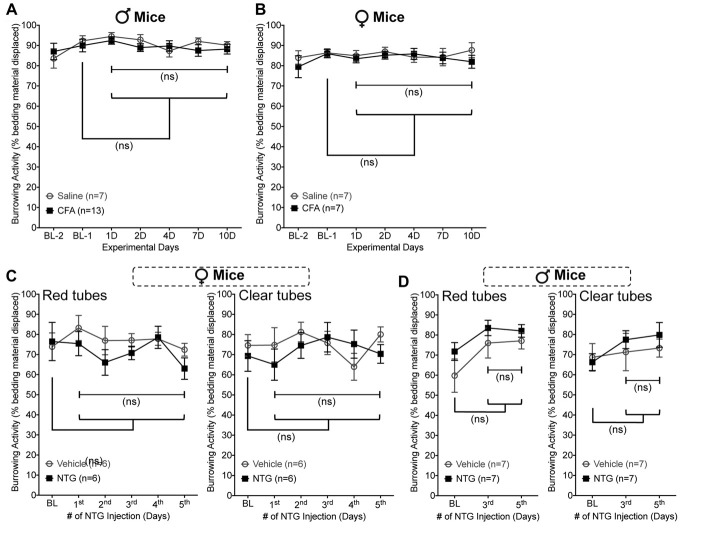
Unilateral Complete Freund’s Adjuvant (CFA) and nitroglycerin (NTG) administration do not suppress burrowing behavior in mice. Burrowing activity is not significantly altered in CFA- (10 μg, i.pl.) vs. saline-injected male **(A)** and female **(B)** mice. Similarly, burrowing activity is unchanged in female **(C)** and male **(D)** mice subjected to nitroglycerin (10 mg/kg, i.p. every other day for five times) injection, irrespective of whether the tubes used were red (left panels) or transparent (right panels). Data are presented as mean ± SEM. “ns”-not significant vs. respective vehicle/saline controls, and experimental days vs. baseline (Bl-1 or BL) values for respective groups, two-way ANOVA with Bonferroni’s *post hoc* correction.

Since SNI is used as a model of neuropathic pain, we wanted to establish whether other pain-related models also lead to suppression of burrowing behavior in mice. Intraplantar (i.pl.) injection of complete Freund’s adjuvant (CFA) is commonly used as a model of inflammatory pain (Ghasemlou et al., 2015). Although CFA was administered unilaterally at a dose which causes thermal and mechanical hypersensitivity in ipsilateral hindpaws and persists over 7–10 days (10 μl at 1 mg/ml, i.pl.; data not shown), no deficit in bedding displacement was observed in male or female mice (Figures 6A,B). The same was also true of systemic administration of nitroglycerin (NTG; 10mg/kg, i.p. every other day for five times), which is used to model migraine-like symptoms and induces persistent referred mechanical hypersensitivity in mice (data not shown; Bates et al., 2010; Pradhan et al., 2014). In female (Figure 6C) and male (Figure 6D) mice, no deficit in bedding displacement was observed in NTG-injected vs. vehicle-injected mice. In addition, since NTG enhances symptoms of photophobia in rodents (Harris et al., 2017), we tested transparent vs. red tubes with both sexes. Again, no significant deficit in burrowing behavior was induced by administration of NTG. Altogether, these findings suggest that the ability of burrowing behavior measurement paradigm to detect pain-related alterations in behavior in mice exist among some pain models, but not others.

### Discussion

Analgesic drug development has been impeded by the types of pre-clinical models of human pain pathologies (Barrot, 2012; Clark, 2016). Historically, assessment of pain sensitivity in rodents has relied upon measures that have an evoked, reflexive end-point, such as latency to tail-flick or hindpaw withdrawal. The development of novel, voluntary, non-reflexive assays of pain behaviors in rodents aims to improve the translation of pre-clinical findings into clinical developments.

The assessment of a rodent’s volition to clear material from an enclosed space was initially performed to explore behavioral deficits associated with central nervous system neurodegeneration (Deacon et al., 2001; Jirkof, 2014). It has since been adapted for assessment of inflammatory and neuropathic pain behaviors in mice and rats, with considerable variation in tube type, burrowing materials and assay duration (Jirkof et al., 2010; Andrews et al., 2012; Huang et al., 2013; Lau et al., 2013; Rutten et al., 2014; Bryden et al., 2015; Gould et al., 2016; Muralidharan et al., 2016; Wodarski et al., 2016; Das et al., 2017). Several different rodent species have been tested in burrowing behavior paradigms in a laboratory setting (Deacon, 2009), with some variability in burrowing performance reported between different mouse strains. C57BL/6J mice were consistently found to burrow more extensively than 129S2/Sv mice, for example (Contet et al., 2001). Our study aimed to characterize the impact of several key variables in a systematic fashion: strain, sex, assay duration, tube color/light intensity and neuropathic vs. inflammatory vs. migraine-based pain models. In contrast to prior studies, our experimental setup generated a level of burrowing that was experimentally useful within 15 min, in male or female mice, using red-tinted tubes (Figure 2). This much shorter protocol is likely to avoid inducing chronic stress in mice due to social isolation, a potential confounding factor in repeated measurement-based studies. This reduced latency to near-complete displacement may be attributable, at least in part, to our use of home cage bedding material as the burrowing substrate, as opposed to quartz sand/gravel or food pellets used in most reports (Andrews et al., 2012; Gould et al., 2016; Muralidharan et al., 2016; Wodarski et al., 2016). The likely reduction in perceived novelty of a tube filled with familiar material might explain the speed with which the mice were observed to clear the tube. Our study did not find strain differences in baseline and injury-related burrowing activities in mice, although expansion of such studies in a wide variety of mouse strains would be required to draw a broad conclusion on the impact of mouse strain differences on burrowing performance. Furthermore, the possibility remains that the greater range of individual burrowing performance in female vs. male mice is due to estrus cycle-related differences in pain sensitivity (Giamberardino et al., 1997; Bradshaw et al., 2000; Craft et al., 2004; Sanoja and Cervero, 2005). Therefore, future in-depth studies are warranted to explore the potential impact of estrus cycle and different hormonal regulations on burrowing activity under naïve and injury (or pain pathological) conditions.

Although the act of engaging in burrowing behavior may itself be rewarding, we must also factor in the inherent light aversion that is common in rodents. This is exemplified by the inconsistent burrowing performance seen when using transparent tubing, with room lights ON, which was absent when the room lights were switched OFF (Figures 2B, 4A,B). Since rodents have dichromatic vision and are relatively insensitive to red-tinted light (Jacobs, 1993; Peirson et al., 2018), we can surmise that accessing a region of reduced light intensity is likely to be a component of the drive to enter the tube.

Although neuropathic condition did depress burrowing behavior in male (Figures 3A, 4A) and female (Figures 3D, 4B) mice, it did so only temporarily when compared to von Frey-based assessment of hindpaw mechanical sensitivity (Figures 3B,E), which is known to persist for several weeks post-surgery (Decosterd and Woolf, 2000; Shepherd and Mohapatra, 2018). We reasoned that the gradual extinction of the SNI-induced deficit in burrowing behavior when assessed daily might be due one or more factors: habituation to the testing environment, a change in motivation to engage in a potentially rewarding behavior, exercise-mediated analgesia (Naugle et al., 2012) or a learned adaptation of burrowing behavior to circumvent “painful” hind limb stimulation. Accordingly, we repeated the experiments in a separate cohort of mice with neuropathic condition, with an inter-test interval of 5 days (Figure 5A), where the burrowing deficit was maintained. We did not test directly for impairment of locomotion as a contributor to SNI-induced reductions in burrowing behavior in this particular study. However, prior evidence indicates that overall distance traveled (Mogil et al., 2010; Urban et al., 2011; Pitzer et al., 2016), as well as spontaneous walking speed are not affected by neuropathic pain (Mogil et al., 2010; Pitzer et al., 2016; Shepherd and Mohapatra, 2018), making such an explanation unlikely. Furthermore, prior studies have shown no significant reduction in activities, such as in-cage wheel running, in mice with SNI surgery (Sheahan et al., 2017). Interestingly, hindpaw CFA injection has been shown to reduce in-cage wheel running activity in mice (Cobos et al., 2012; Sheahan et al., 2017). Our findings suggest no significant alteration in burrowing activity in mice with hindpaw CFA injection. Altogether, these observations suggest that changes in locomotor activity has limited impact on burrowing behavior in mice.

The correlation between suppression of burrowing and evoked mechanical hypersensitivity (Figures 3C,F), along with the reversal of depressed burrowing by gabapentin (Figure 5B), strongly indicate that burrowing could be considered a pain-depressed behavior. However, both inflammatory pain in the hind paw induced by CFA (Figures 6A,B), and induction of migraine-like symptoms with NTG (Figures 6C,D) did not elicit any change in bedding displacement behavior. This is in contrast to prior studies in rats where a decrease in burrowing following unilateral intraplantar CFA was observed (Andrews et al., 2012; Gould et al., 2016; Muralidharan et al., 2016; Wodarski et al., 2016). This could be attributable to an inherent difference between mice and rats in terms of the sensitivity of burrowing behavior to pain. Alternatively, this may be a dose-related issue; our study administered 10 μg of CFA to a mouse, vs. 100–150 μg in rats. Also, the use of material other than home cage bedding in the burrowing tube, particularly one that requires a greater degree of effort to displace from the tube, may also uncover differences in behavior. Alternatively, the burrowing assay as employed here may simply be a less sensitive assessment of sensory hypersensitivity than more traditional, reflexive end-point assays. Further testing and validation is required to completely address these apparent discrepancies. An alternate explanation on the lack of burrowing behavior changes in CFA and NTG injections vs. SNI in mice could be the chronicity of pain-like hypersensitivity in these models. CFA- and NTG-induced reflexive measures of pain hypersensitivity, and CFA-induced deficits in in-cage wheel running activities in mice have been shown to resolve by 7–10 days post-injection (Bates et al., 2010; Pradhan et al., 2014; Ghasemlou et al., 2015; Shepherd and Mohapatra, 2018). However, in nerve injury rodent models, such as in SNI and CCI, the intensity of pain-related behaviors are sustained for a longer duration, up to several months (Decosterd and Woolf, 2000; Sheahan et al., 2017), which could provide another potential explanation on deficits in burrowing behavior under neuropathic conditions. Also, the potential influence of depression-like behavior associated with mouse models of chronic neuropathic pain (Norman et al., 2010) on their burrowing behavior could not be ruled out. Further in-depth studies are thus needed to validate burrowing behavior in multiple pain-producing pathologies, such as bone cancers, non-surgical forms of neuropathy and inflammation.

Since non-reflexive assays necessarily invoke more complex neurological circuits than reflexive assays, development of a voluntary behavioral assessment of pain that retains the low variability, high sensitivity and widespread applicability of more traditional, un-evoked assays may be extremely difficult to achieve. Instead, a study design that incorporates the strengths of both approaches may be a more optimal approach to acquire pre-clinical data that ultimately translate into new therapeutic opportunities.

## Author Contributions

DM performed the design and fabrication of the burrowing tubes. AS, MC and DM performed experiments and analyzed the data. DM and AS designed the experimental protocols for neuropathic and inflammatory pain mouse models. MC and Y-QC designed the experimental protocol for mouse migraine model. AS and DM wrote the manuscript with input from MC and Y-QC.

## Conflict of Interest Statement

The authors declare that the research was conducted in the absence of any commercial or financial relationships that could be construed as a potential conflict of interest.
